# Field Evaluation of a Push-Pull System to Reduce Malaria Transmission

**DOI:** 10.1371/journal.pone.0123415

**Published:** 2015-04-29

**Authors:** David J. Menger, Philemon Omusula, Maarten Holdinga, Tobias Homan, Ana S. Carreira, Patrice Vandendaele, Jean-Luc Derycke, Collins K. Mweresa, Wolfgang Richard Mukabana, Joop J. A. van Loon, Willem Takken

**Affiliations:** 1 Laboratory of Entomology, Wageningen University, P.O. Box 8031, 6700 EH, Wageningen, The Netherlands; 2 International Centre of Insect Physiology and Ecology, P.O. Box 30772, GPO Nairobi, Kenya; 3 CIEPQPF, Department of Chemical Engineering, University of Coimbra, Rua Sílvio Lima, 3030–790, Coimbra, Portugal; 4 Devan—Micropolis, Tecmaia-Parque da Ciência e Tecnologia da Maia, Rua Eng. Frederico Ulrich, 2650, 4470–605, Maia, Portugal; 5 Devan Chemicals NV, Ninoofsesteenweg 539, 9600, Ronse, Belgium; 6 Utexbel NV, C. Snoecklaan 30, B-9600, Ronse, Belgium; 7 School of Biological Sciences, University of Nairobi, P.O. Box 30197–00100, GPO Nairobi, Kenya; University of Crete, GREECE

## Abstract

Malaria continues to place a disease burden on millions of people throughout the tropics, especially in sub-Saharan Africa. Although efforts to control mosquito populations and reduce human-vector contact, such as long-lasting insecticidal nets and indoor residual spraying, have led to significant decreases in malaria incidence, further progress is now threatened by the widespread development of physiological and behavioural insecticide-resistance as well as changes in the composition of vector populations. A mosquito-directed push-pull system based on the simultaneous use of attractive and repellent volatiles offers a complementary tool to existing vector-control methods. In this study, the combination of a trap baited with a five-compound attractant and a strip of net-fabric impregnated with micro-encapsulated repellent and placed in the eaves of houses, was tested in a malaria-endemic village in western Kenya. Using the repellent delta-undecalactone, mosquito house entry was reduced by more than 50%, while the traps caught high numbers of outdoor flying mosquitoes. Model simulations predict that, assuming area-wide coverage, the addition of such a push-pull system to existing prevention efforts will result in up to 20-fold reductions in the entomological inoculation rate. Reductions of such magnitude are also predicted when mosquitoes exhibit a high resistance against insecticides. We conclude that a push-pull system based on non-toxic volatiles provides an important addition to existing strategies for malaria prevention.

## Introduction

Malaria continues to place a substantial burden on people throughout the tropics and especially in sub-Saharan Africa. Current prevention efforts focus on long-lasting insecticidal nets (LLINs) and indoor residual spraying (IRS) to control mosquito populations [1]. Although these measures have led to significant decreases in the number of malaria cases, progress is threatened by the development and rapid spread of insecticide-resistance [2,3]. An additional threat is the shift from indoor to outdoor feeding as well as changes in biting times that have been observed following the implementation of ITNs and IRS [4–7]. Furthermore, changes in the composition of vector populations may lead to the dominance of species with a different ecology, which are harder to target with conventional approaches [8,9].

A mosquito-directed push-pull system, which operates by the simultaneous use of attractant and repellent cues, offers a possible alternative or addition to current vector control methods [10–12]. Previous experiments have shown that a push-pull system employing attractant-baited traps and spatial repellents can be effective at lowering the house entry of malaria mosquitoes by as much as 95% in an experimental setup [12]. Residents would receive considerable protection by such a reduction in mosquito exposure.

Thus far, however, all research concerning this type of push-pull system for malaria mosquitoes has taken place under semi-field conditions. For practical implementation of the system in rural Africa it is important that the system be low-tech and not dependent on electric power. This would involve limiting the number of attractant-baited traps and finding an alternative for electric power-dependent systems for the dispersal of repellents. Moreover, the system should be designed in such a way that it can run independently for a prolonged period of time.

Recent large-scale field studies are exploring the potential of mass-trapping of mosquitoes by employing a single attractant-baited trap per household that can run on solar power [13]. Supplementing this with a passive (i.e. not requiring energy input) repellent release mechanism would provide a push-pull system that is both user-friendly and practical for real-world implementation.

Impregnated textile fabrics can be employed as suitable materials for passive dispersion of repellents [14]. Durable textiles can also be used for eave-screening, providing a combination of two efficient mechanisms by creating a physical as well as a chemical mosquito barrier. A prolonged passive release of repellent compound can be achieved by using a microencapsulation technique [15]. Microcapsules can be impregnated into many different kinds of fabric and offer a novel method to control the release of active compounds. This technique makes it possible to obtain a longer lasting repellent effect than when the active compound is directly applied to the textile [16,17].

In the present study we first determined the longevity of the repellent effect of a fabric that was impregnated with porous microcapsules containing delta-undecalactone, a compound which has recently been shown to have strong repellent properties against several mosquito vectors of disease [12,18]. Subsequently we deployed a push-pull system that uses a trap baited with a five-compound attractive blend + CO_2_ [12] in combination with this repellent-impregnated fabric in a malaria-endemic village in western Kenya. We explored the possible effects of large-scale application of the described push-pull intervention on human-mosquito contact and malaria transmission by adapting an existing mathematical model.

## Results

### Laboratory experiment

**Fig 1:**
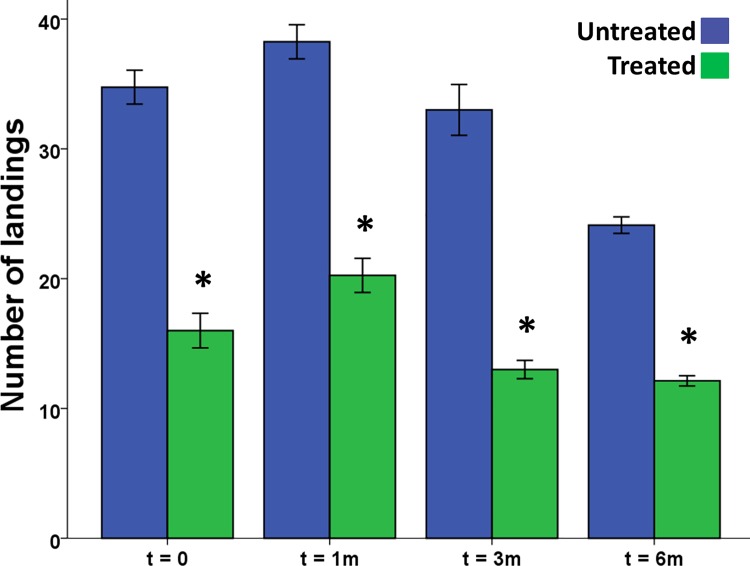
Mean number of mosquito landings on the control and the treated fabrics. At zero, one, three and six months after treatment. Asterisks indicate a significant difference between the control and the treatment, n = 8 for all groups, error bars indicate the standard error of the mean.

Experiments were conducted in the set up described by Menger et al. [18], in which mosquitoes (*Anopheles coluzzii*, formerly *An*. *gambiae s*.*s*. form M) were given the opportunity to land on an artificial bait. At all tested times, t = 0, t = 1 month, t = 3 months and t = 6 months, a significant repellent effect was found for fabric impregnated with microencapsulated delta-undecalactone (Independent Samples t-test, p < 0.001 for all comparisons; Fig 1). The reduction in the number of landings was similar (ranging from 47 to 61%) at all tested time points.

### Field experiment

Four treatments were tested in Kigoche village in Kisumu county, western Kenya: (i) the control treatment, in which a house received neither repellent-impregnated fabric nor an attractant-baited trap. (ii) a push-only treatment in which only the repellent-impregnated fabric was installed, (iii) a pull-only treatment in which an attractant-baited MM-X trap was installed outside the house and (iv) a push-pull treatment in which both the repellent-impregnated fabric and the attractant-baited trap were in place. For the duration of the experiment, houses were occupied by one male volunteer only, who slept under an untreated bed net. The house entry rate of mosquitoes was determined by CDC light trap catches [19,20]. Preceding the experiment, a baseline study was carried out in order to be able to correct for randomization bias, as treatments were not to be rotated between houses because of possible residual effects.

During the entire experiment, 1,791 mosquitoes were caught inside the houses (96.9% female, 3.1% male) of which 1,724 (96.3%) were anophelines and 67 (3.7%) culicines. The anopheline population consisted of 80.2% *An*. *funestus* s.l. and 19.8% *An*. *gambiae* s.l. A sub-sample of 188 individuals of *An*. *funestus* was molecularly studied for sub-species composition [21,22]. The 177 samples that were successfully amplified were all *An*. *funestus s*.*s*. Out of 184 *An*. *gambiae* individuals that were analysed molecularly [23], 171 were successfully amplified and all were *An*. *arabiensis*.

Statistical analyses were done for the overall CDC trap catches and for the anopheline sub-group, other sub-groups were considered too small to carry out reliable statistics, but their values are reported below and more details can be found in S1 and S2 Tables in the supporting information.

The four houses that were selected for the intervention from the baseline study were the ones that were most similar in terms of mean trap catches and variation over the subsequent nights (Table 1). Within the five-week intervention phase, there was no increase or decrease in trap catches as a function of time (GLM with overall CDC trap catches as dependent variable, ‘intervention’ as a fixed factor and ‘week’ as a covariate, full-factorial: p = 0.001 for intervention, p = 0.629 for week and p = 0.711 for intervention*week). Therefore the samples over the whole intervention period were pooled, resulting in 25 replicate measurements for each group.

**Table 1:** Mean number (+SD) of mosquitoes caught during the baseline phase.

House	Baseline		Intervention
** **	**Mean**	**SD**	** **
1	21.75	6.94	Push-pull
2	6.63	3.74	not selected
3	11.00	5.23	Pull
4	15.75	4.30	Control
5	14.00	7.09	Push
6	6.25	6.82	not selected
7	21.63	14.26	not selected
8	7.88	3.87	not selected

For all houses n = 8, except for house 3 (n = 7). Four houses were selected for the different interventions.

**Fig 2:**
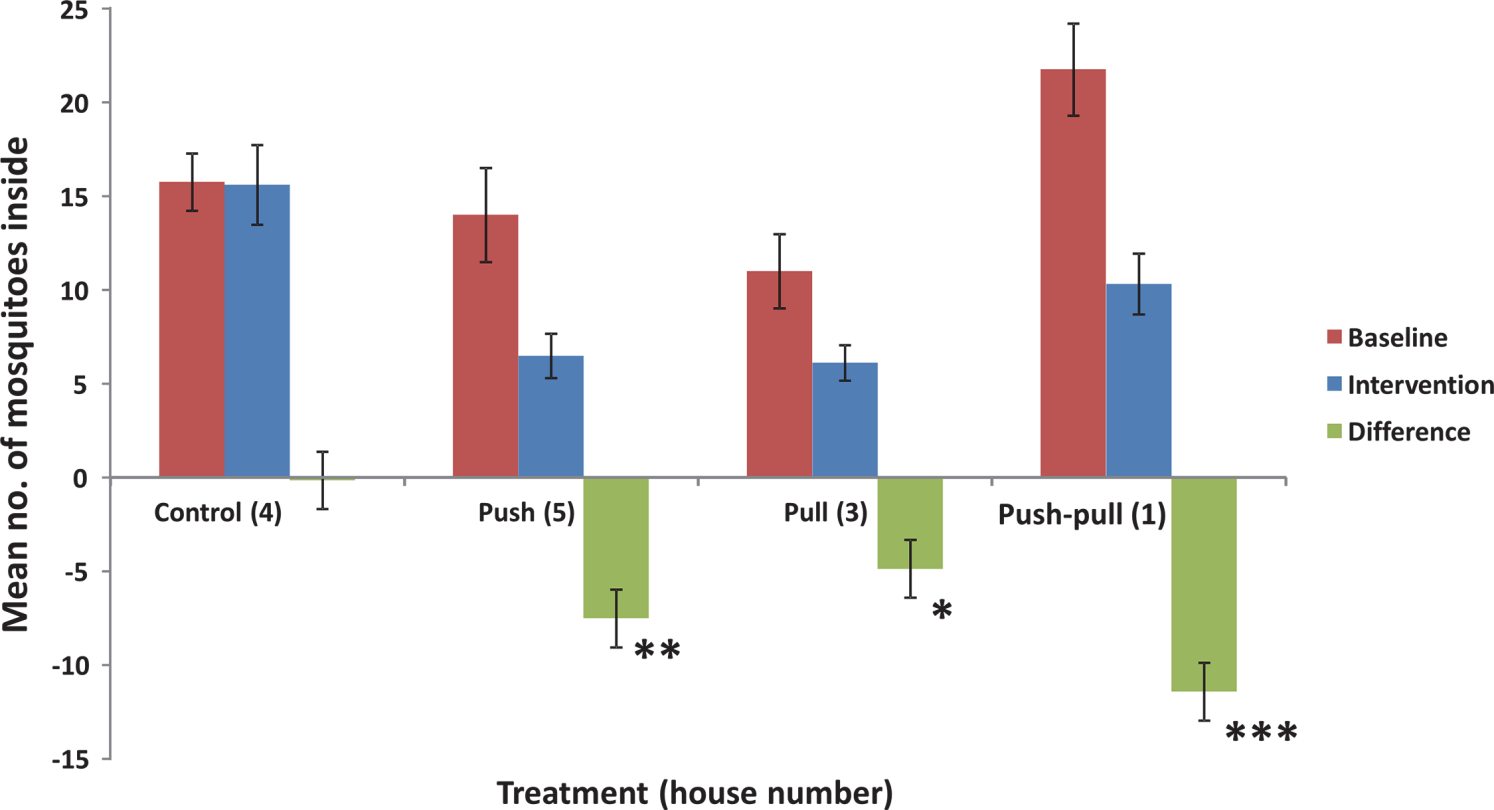
Mean number of mosquitoes caught inside the houses. Error bars indicate standard error of the mean (SEM), n = 8 for the baseline data (n = 7 for house 3) and n = 25 for the intervention data. Asterisks indicate a significant difference-in-differences between the control and the intervention: * p < 0.05; ** p < 0.01; *** p < 0.001.

Significant reductions in house entry of mosquitoes were found for all interventions (Fig 2). The push-only intervention reduced mosquito house entry by 52.8% compared to the control. The pull-only intervention reduced mosquito house entry by 43.4% and the push-pull intervention reduced mosquito house entry by 51.6% (Table 2).

**Table 2:** Mean overall CDC trap mosquito catches for the different interventions.

Intervention	House	Baseline	Intervention	Difference	Difference (%)	Impact
Control	4	15.75	15.60	-0.15	-1.0%	n/a
Push	5	14.00	6.48	**-7.52	-53.7%	-52.8%
Pull	3	11.00	6.12	*-4.88	-44.4%	-43.4%
Push-pull	1	21.75	10.32	***-11.43	-52.6%	-51.6%

For the baseline data n = 8 (n = 7 for house 3) and n = 25 for the intervention data. Asterisks indicate a significant difference-in-differences between the control and the intervention

* p < 0.05

** p < 0.01

*** p < 0.001.

**Fig 3:**
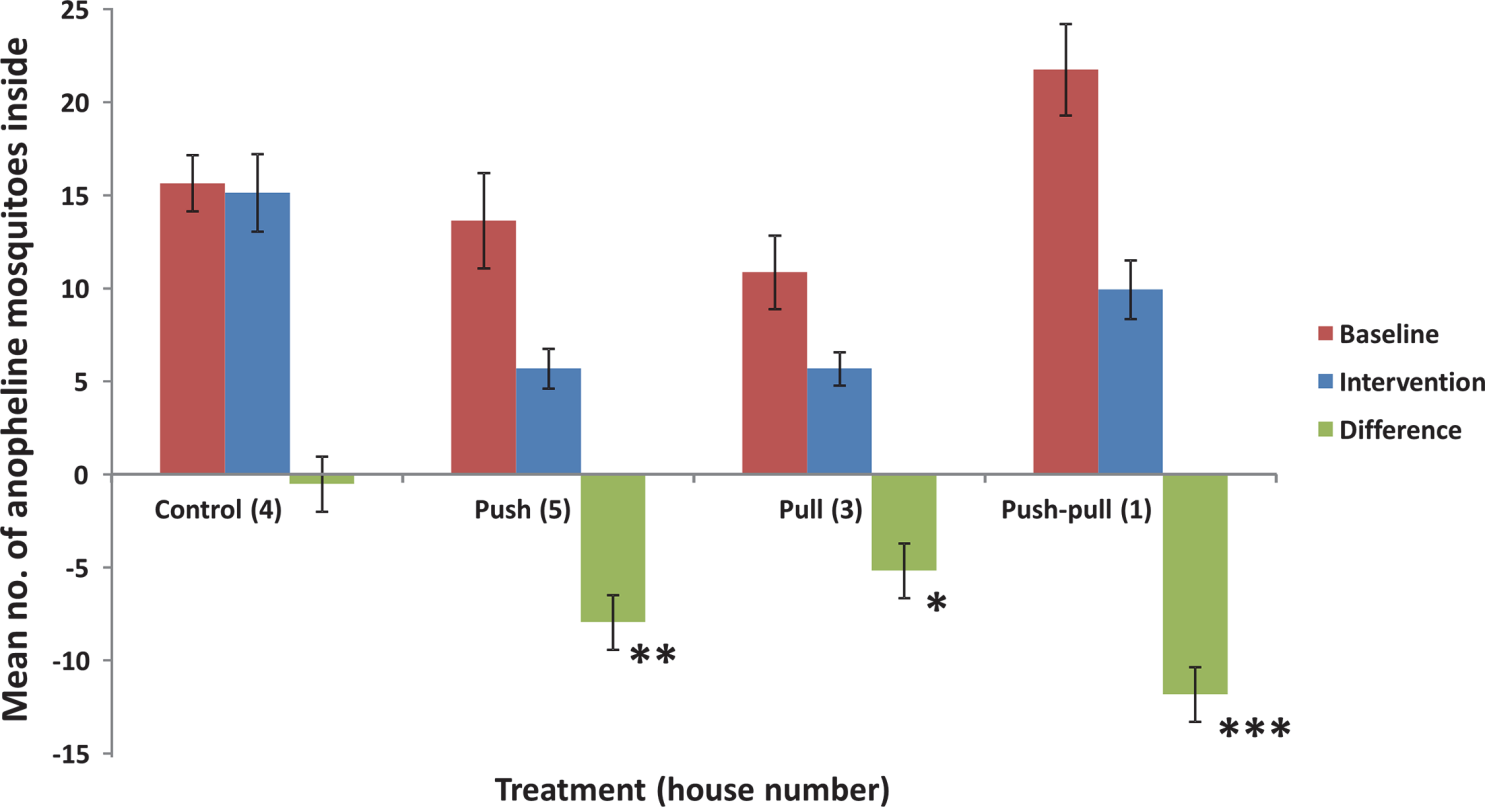
Mean number of anopheline mosquitoes caught inside the houses. Error bars indicate standard error of the mean (SEM), n = 8 for the baseline data (n = 7 for house 3) and n = 25 for the intervention data. Asterisks indicate a significant difference-in-differences between the control and the intervention: * p < 0.05; ** p < 0.01; *** p < 0.001.

Considering anopheline mosquitoes only, the results were fairly similar, with all interventions resulting in significant reductions in house entry (Fig 3). The impact of the different interventions was 55.1% for the push-only, 44.4% for the pull-only and 51.1% for the push-pull intervention (Table 3). For *An*. *funestus*, house entry reductions were 59.5, 47.4 and 48.9% for the push-only, pull-only and push-pull interventions, respectively (S1 Table for more details). House entry reductions for *An*. *gambiae* s.l. were 32.9, 29.3 and 39.0% respectively (S2 Table). No further calculations were done for the *Culex* and *Mansonia* subgroups, as the low numbers of caught individuals (58 and 9 in total, respectively) would not allow us to draw reliable conclusions.

**Table 3:** Mean CDC trap catches of anopheline mosquitoes for the different interventions.

Intervention	House	Baseline	Intervention	Difference	Difference (%)	Impact
Control	4	15.63	15.12	-0.51	-3.3%	n/a
Push	5	13.63	5.68	**-7.95	-58.3%	-55.1%
Pull	3	10.86	5.68	*-5.18	-47.7%	-44.4%
Push-pull	1	21.75	9.92	***-11.83	-54.4%	-51.1%

For the baseline data n = 8 (n = 7 for house 3) and n = 25 for the intervention data. Asterisks indicate a significant difference-in-differences between the control and the intervention

* p < 0.05

** p < 0.01

*** p < 0.001.

The MM-X traps placed outdoors in the pull-only and push-pull treatments caught 1,356 mosquitoes (95.6% female, 4.4% male) in total, of which 616 (45.4%) were anophelines and 740 (54.6%) culicines. The anophelines were 52.1% *An*. *funestus*, 43.8% *An gambiae* s.l. and 4.1% other anopheline spp. The mean number of mosquitoes caught outside in the push-pull treatment (29.16, SEM 4.32) was not significantly different from the mean number caught in the pull-only treatment (25.08, SEM 2.54).

### Malaria transmission model

To simulate the effect of implementation of the push-pull strategy on a large scale, we adjusted an existing mathematical model by Okumu et al. [24]. We used the default settings of the model, with exceptions for: bed net use (Ch), which was set at 67%; human availability (ah), which was translated to relative human availability (rah) to model the effect of house entry reduction (expressed as push efficacy: ps) and; attractiveness of the attractant-baited traps (λt). The effects of possible push-pull interventions in a situation in which pyrethroid resistance is widespread (reducing excess mosquito mortality (θm)) was explored in a second scenario.

**Fig 4:**
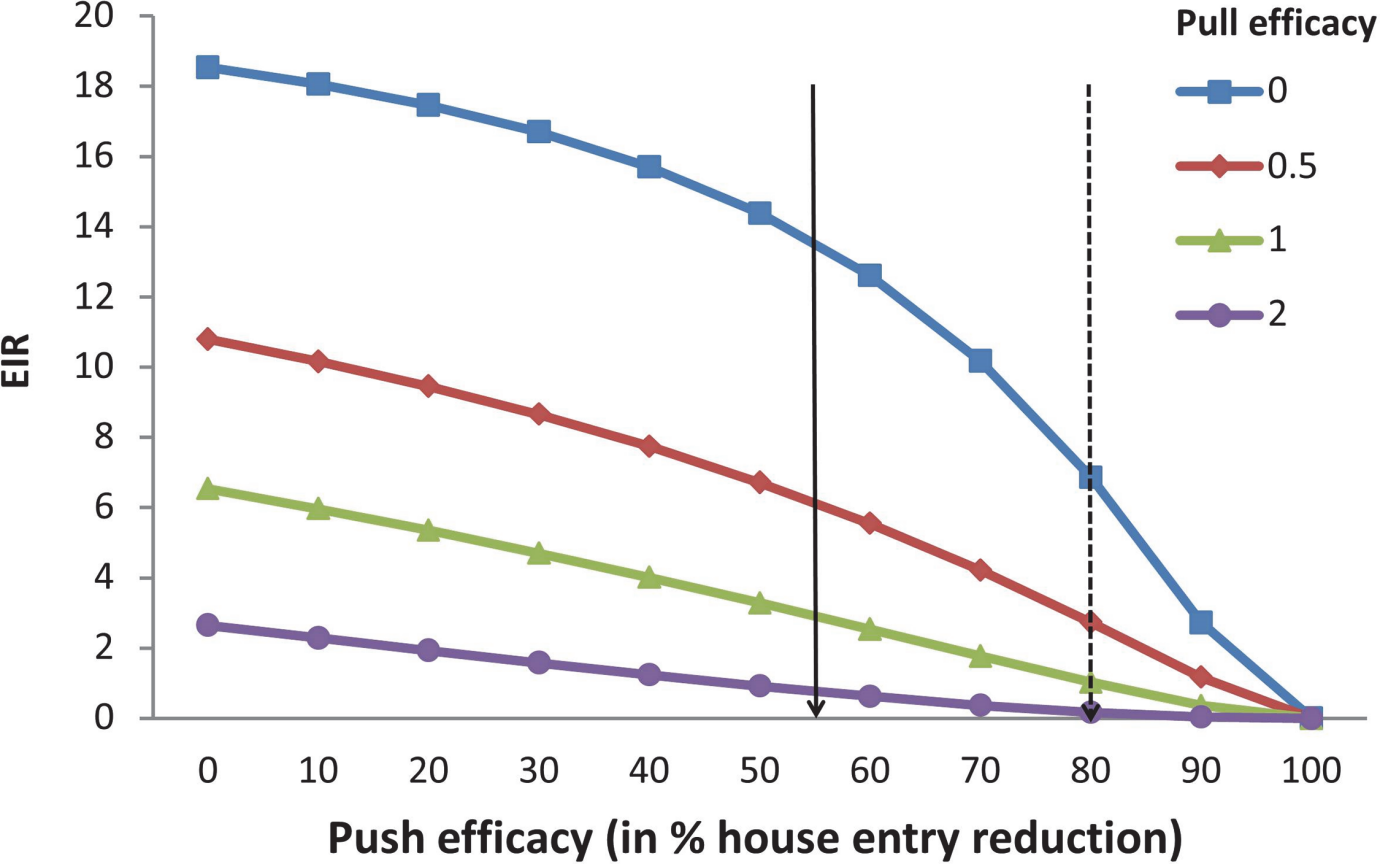
Model simulations showing the entomological inoculation rate (EIR) as a function of different levels of push efficacy. Push efficacy is expressed as the percentage of house entry reduction and pull efficacy is expressed as the relative attractiveness of the trap, compared to a human being. In this scenario mosquitoes are fully susceptible to insecticides.

**Fig 5:**
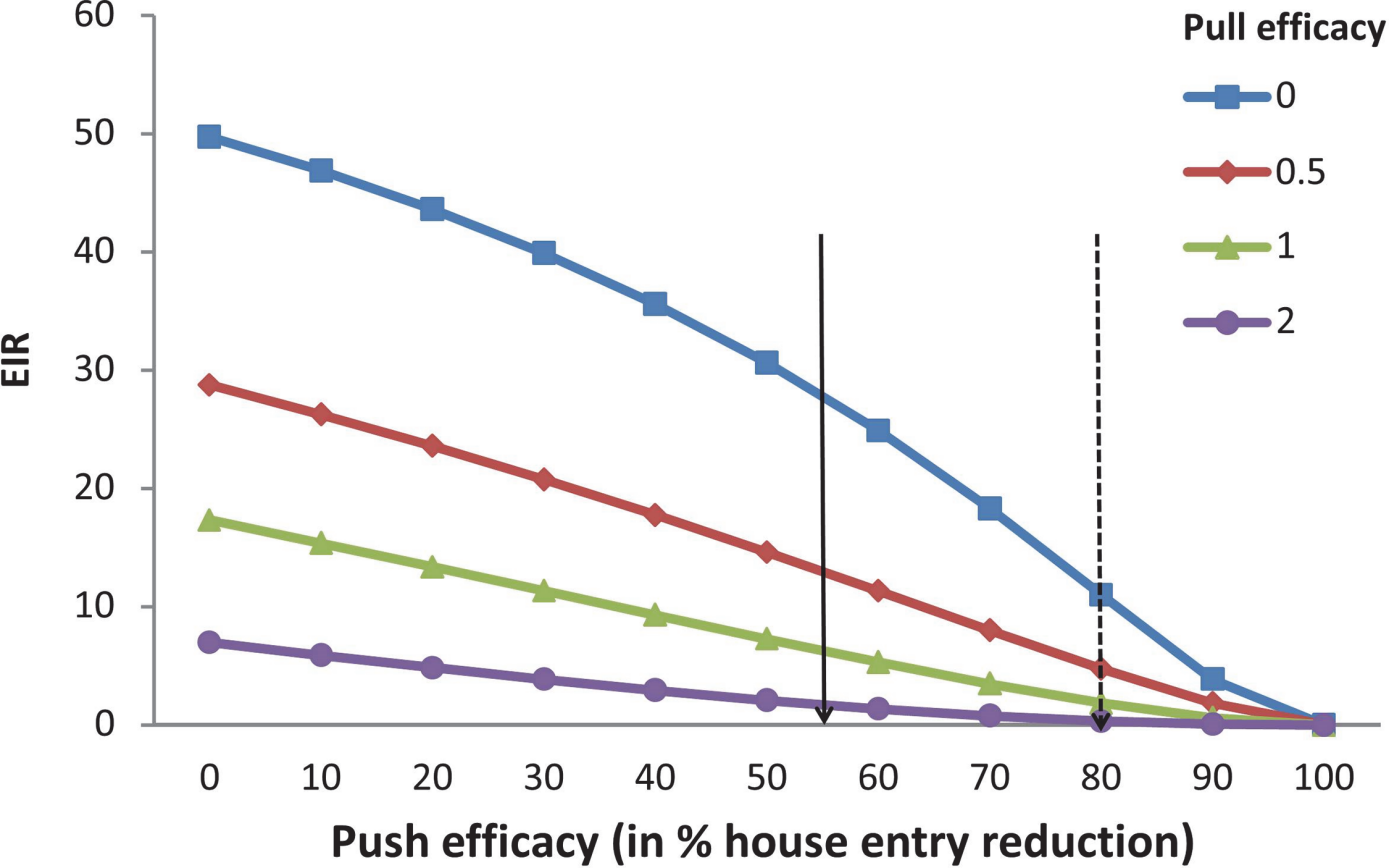
Model simulations of a scenario in which mosquitoes are highly resistant against insecticides. Shown is the entomological inoculation rate (EIR) as a function of different levels of push efficacy. Push efficacy is expressed as the percentage of house entry reduction and pull efficacy is expressed as the relative attractiveness of the trap, compared to a human being.

Model simulations predict the impact of a large-scale push-pull intervention on the EIR (Figs 4 and 5). Under the given assumptions, either repellent barriers or odour-baited traps alone result in evident reductions of the EIR. However, the strongest reductions are obtained when combining push and pull.

In the first scenario, assuming 67% ITN coverage and susceptible mosquitoes, the initial EIR is estimated at 18.5 infectious bites per year (Fig 4, see also S1 Fig). Combining a repellent barrier with a push efficacy of 55% reduction in house entry (as was found in this study and is indicated with a solid arrow in Fig 4) with an odour-baited trap that has the same attractiveness as a human being [25,26] (the green line / triangles in Fig 4) would reduce the EIR to 2.9 infectious bites per year. If the push efficacy can be improved to 80% (which is deemed feasible by screening the eave entirely with repellent material and is indicated with a dotted arrow in Fig 4), the combination with a trap reduces the EIR to 1.0 in our model (a nearly 20-fold reduction). The repellent barrier only reduces the EIR to 13.5 or 6.9 when the push efficacy is 55 or 80%, respectively. Attractant-baited traps alone are estimated to reduce the EIR to 6.5, if the attractiveness of the traps is the same as that of a human being.

In the second scenario, mosquitoes are assumed to have high resistance against the insecticides used on ITNs. The EIR is calculated to be much higher, with an initial value of 49.7 infectious bites per year (Fig 5, see also S2 Fig). In this case a repellent barrier with a push efficacy of 55% reduction in house entry (solid arrow) that is combined with an odour-baited trap that has the same attractiveness as a human being (green line / triangles) reduces the EIR to 6.3 infectious bites per year. In case the push efficacy can be improved to 80%, the combination with a trap is predicted to reduce the EIR to 1.9 (a more than 20-fold reduction). The repellent barrier in the absence of a trap reduces the EIR to 27.8 or 11.0 when the push efficacy is 55 or 80%, respectively. Attractant-baited traps alone, having the same attractiveness as a human being, are predicted to reduce the EIR to 17.3.

## Discussion

### Interpretation of results

This study showed how repellent-treated fabrics, whether or not in combination with an attractant-baited trap, reduced mosquito house entry by approximately 50% in a field setting. Model simulations predict that when a push-pull intervention is applied on a large scale, up to 20-fold reductions in the EIR may be obtained by combining the repellent fabric and attractant-baited traps.

Behavioural tests in the repellent bioassay showed a consistent repellent effect of the treated fabric, which was maintained for a period of at least six months. Because samples were stored in plastic bags in a refrigerator in between the tests, evaporation of volatiles from the fabric was presumably much lower than under field circumstances. The fabric intended for use in the field study was prepared identically and stored for two months in the same way, thus we expect it to have been similarly efficient by the time it was applied in the field.

During the field experiment, we found that even a 10 cm wide strip of this repellent-treated fabric reduced mosquito house entry by 52.8%. This must have resulted from a ‘barrier’ of repellent, as the fabric did not physically close off the eave, leaving ample space for mosquitoes to fly over as they did in the control treatment with untreated fabric. Mnyone et al. [27] similarly closed off the eaves partially with baffles, and demonstrated that such imperfect barriers do not affect house entry of *An*. *gambiae* s.l. and *An*. *funestus*. Snow [28] reported that endophilic host-seeking mosquitoes fly towards a human-occupied house (presumably following a CO_2_ gradient [29,30]) where, upon reaching a vertical wall, they fly upwards until entering through the eave. For this reason, we chose to apply the fabric to the lower part of the eave, closing off the bottom 10 cm rather than the middle or upper section, to make sure that mosquitoes would encounter the fabric before entering the house.

The employment of an attractant-baited trap outside the experimental house reduced mosquito house entry by 43.4%. This suggests that mosquitoes were lured into the trap before they could enter the house. This is an unexpected result, as previous observations indicated that outdoor traps do not directly influence mosquito house entry [30]. However, the positioning of the trap, relative to the location of mosquito breeding sites or resting places, may potentially influence the trap’s efficacy in luring mosquitoes away from a house before entering [31]. Moreover, the outdoor trap caught 25 mosquitoes per night on average, which is a considerably higher number than the 6 individuals caught by the CDC trap indoors during the pull-only intervention or the 16 individuals that were caught on average in the control house. Although these catches cannot be compared directly since different trapping methods were used indoors and outdoors, it confirms findings from previous studies showing that attractant-baited traps are a very potent tool to remove large numbers of mosquitoes [25,26].

When the repellent-treated fabric and the attractant-baited trap were combined, mosquito house entry was reduced by 51.6%. This reduction is a bit higher than the reduction achieved by the attractant-baited trap alone, but rather similar to the reduction achieved by the repellent alone. This result may seem surprising, but is actually in line with our earlier conclusion that ‘rather than a synergetic interaction, both components (i.e. the push and the pull) seem to have independent effects’ [12]. It could thus be concluded that there is no additive effect of the attractant-baited trap and that the repellent-treated fabric, which has the higher impact and is also much cheaper, should be the recommended intervention.

Model simulations however, show that when all households are covered by the intervention, malaria transmission is reduced most effectively by augmenting the existing prevention efforts with the complete push-pull system. Whereas both the repellent barrier and the attractant-baited trap reduce the EIR independently, it is their combination that causes the strongest (up to 20-fold) reductions. This shows that the short term effects of a limited number of traps, as measured during the field experiment, may greatly differ from the effect of large-scale deployment of traps over a longer period of time, as simulated in the model. In the push-pull intervention of our experiment, an average of 29 mosquitoes per night were caught in the outdoor trap, compared to 10 mosquitoes by the trap indoors. Large-scale deployment of traps that catch such high numbers of mosquitoes is expected to affect malaria transmission by reducing the mosquito’s lifespan and by depleting mosquito populations [13,32]. It is because of this indirect effect, that simultaneous deployment of the attractant and repellent may still lead to a greater impact on the EIR, not through a synergism, but rather through complementary functions. Especially in the high insecticide-resistance scenario, it is the combination of the repellent barrier and attractant-baited traps that is able to bring the EIR down to values that would drastically reduce malaria transmission.

The required efficacy of the push and the pull components lies within the range of what has experimentally been shown to be feasible. For example, a repellent barrier with an efficacy of 55% has been found in this study for the house entry of anopheline mosquitoes. This efficacy could most likely be improved by closing off much more of the eave, instead of leaving most of it open (as was done here for experimental purposes). In a previous study in a semi-field setup, house entry was reduced by 80% using only a repellent [12]. Odour baits with an attractiveness similar to that of humans have already been identified [25,26] and are currently being deployed in a large field trial [13].

The dominant malaria vector trapped indoors was *An*. *funestus* (80% versus 20% *An*. *arabiensis*). This is in line with the acknowledgment of *An*. *funestus* as an anthropophilic and endophilic vector, whereas *An*. *arabiensis* is a much more opportunistic feeder that may attack cattle as well as humans, indoors or outdoors [33]. Indeed, in the outdoor traps, the proportion of *An*. *arabiensis* was much higher (44%) and closer to the proportion of *An*. *funestus* (52%). Whereas the repellent barrier is expected to affect mainly indoor transmission, outdoor traps may have an impact on indoor as well as outdoor transmission, because they target vector species with diverse host-seeking behaviours. Further studies should elucidate the functioning of the respective push and pull components in more detail (e.g. the spatial range of the repellent barrier and the optimal placement of the attractant-baited trap relative to it).

### Push-pull as a vector-control tool

In our study, fabrics treated with delta-undecalactone reduced mosquito house entry. When implemented as a vector-control tool, one would not use narrow strips of fabric that leave open most of the eave for mosquitoes to enter, as was done in this study for experimental purposes. Rather, one would close off all openings as much as possible, to install a physical barrier, in addition to the semiochemical one. This of course, brings to mind the practise of screening eaves and/or ceilings, which has already proven to be an effective measure against mosquito house entry [34–36]. However, house screening is difficult in the typical mud-walled houses that make up the majority of houses in the village in which this study was conducted, or indeed in many other traditional hand-built houses that are commonly found in the African countryside. The many cracks and uneven edges hinder the complete closure of the eave, or other openings, with gauze or netting. However, eave screens which are impregnated with a long lasting spatial repellent would not need to close off each little hole and crack as they would serve as a semiochemical barrier as well. Furthermore, net fabric made of cotton is cheap, readily available and allows some degree of air circulation, the main purpose of eaves.

Field experiments employing a repellent to reduce house entry are many, but few report effects of the magnitude observed in this study for a prolonged period of time (i.e. more than a few hours) [37]. One category of repellents that do cause very significant reductions in house entry are the volatile pyrethroids [38,39]. Application of these volatile, or vaporized insecticides resulted in house entry reductions of over 90% in houses with open eaves or similar constructions. However, there are two main objections against the use of insecticides. The first is the development of physiological and behavioural resistance in the target species [2,40]. Although to repel mosquitoes is not the same as to kill them, and thus may be less prone to the development of resistance, these chemicals are from the same class, the pyrethroids, as those used on bed nets (which are meant to kill) and structurally similar. The second, but no less important, argument against pyrethroid insecticides is the concern about the health effects on humans who are exposed to the chemical for prolonged periods of time [41]. A volatile insecticide, dispensed in or around human dwellings would be inhaled, increasing one’s exposure to potentially harmful chemicals. Delta-undecalactone is a natural product that is present in food sources such as edible fruits and dairy products and its odour is generally described as fruity, coconut-like and pleasant [42,43].

In the system presented here, the push and the pull component appear to operate independently. In other words, mosquitoes that are pushed away from the house, do not have a greater chance of being pulled into the trap. This may actually be an advantage, as it would decrease the chance that mosquitoes develop insensitivity to the repellent, which would be stimulated if mosquitoes that are pushed away would have a greater chance of dying in a trap. However, this observation would have to be confirmed in a larger field study, as in the current situation mosquitoes that were repelled may have been diverted to surrounding houses that did not receive the intervention [44]. Therefore, a field study in which a majority of houses in the area receives the push-pull intervention, and all houses are monitored, is a recommended next step.

Based on model simulations, we expect that in a scenario in which coverage of the intervention is high, the greatest benefit can be gained by using both repellent barriers and odour-baited trapping devices to reduce malaria transmission. An advantage of using an odour-baited trap next to a repellent is that mosquitoes are not only repelled from a house, but also actively removed by the trap. As previously shown for trypanosomiasis (sleeping sickness) and other vector-borne infectious diseases, baited traps can be a very efficient tool to lower vector populations and reduce transmission [31,32]. As the odour-bait is a blend that consists of five different compounds, all of which are also present in human skin emanations, it is unlikely that mosquitoes would rapidly become insensitive to it.

In conclusion, the push-pull system based on attractive and repellent volatiles seems a promising addition to the repertoire of integrated vector management, as it may contribute strongly to malaria prevention. It is expected to add to the effect of existing methods such as ITNs and IRS, especially in areas where insecticide resistance is widespread and in situations where malaria transmission occurs outdoors. Its efficacy to reduce malaria transmission should be confirmed in larger-scale field experiments, preferably in combination with existing vector-control tools.

## Materials and Methods

### Components of the push-pull system

#### Attractant

A five-compound odour bait, which simulates human scent, was used as an attractant in both the laboratory and field experiments [18,26,45]. In the laboratory experiment, it provided baseline attraction against which the activity of candidate repellents could be measured. In the field experiment the odour bait was used in combination with CO_2_ to bait the mosquito traps. The blend consists of ammonia, L-(+)-lactic-acid, tetradecanoic acid, 3-methyl-1-butanol and butan-1-amine. Individual compounds were released from nylon strips in concentrations optimized for this release method [46,47].

#### Repellent

The repellent used in this study was delta-undecalactone, a novel repellent which has been shown to be effective against *An*. *coluzzii*, *An*. *gambiae* and *Aedes aegypti* mosquitoes in laboratory and semi-field setups [12,18]. The repellent was released from microcapsules incorporated into cotton netting.

**Fig 6:**
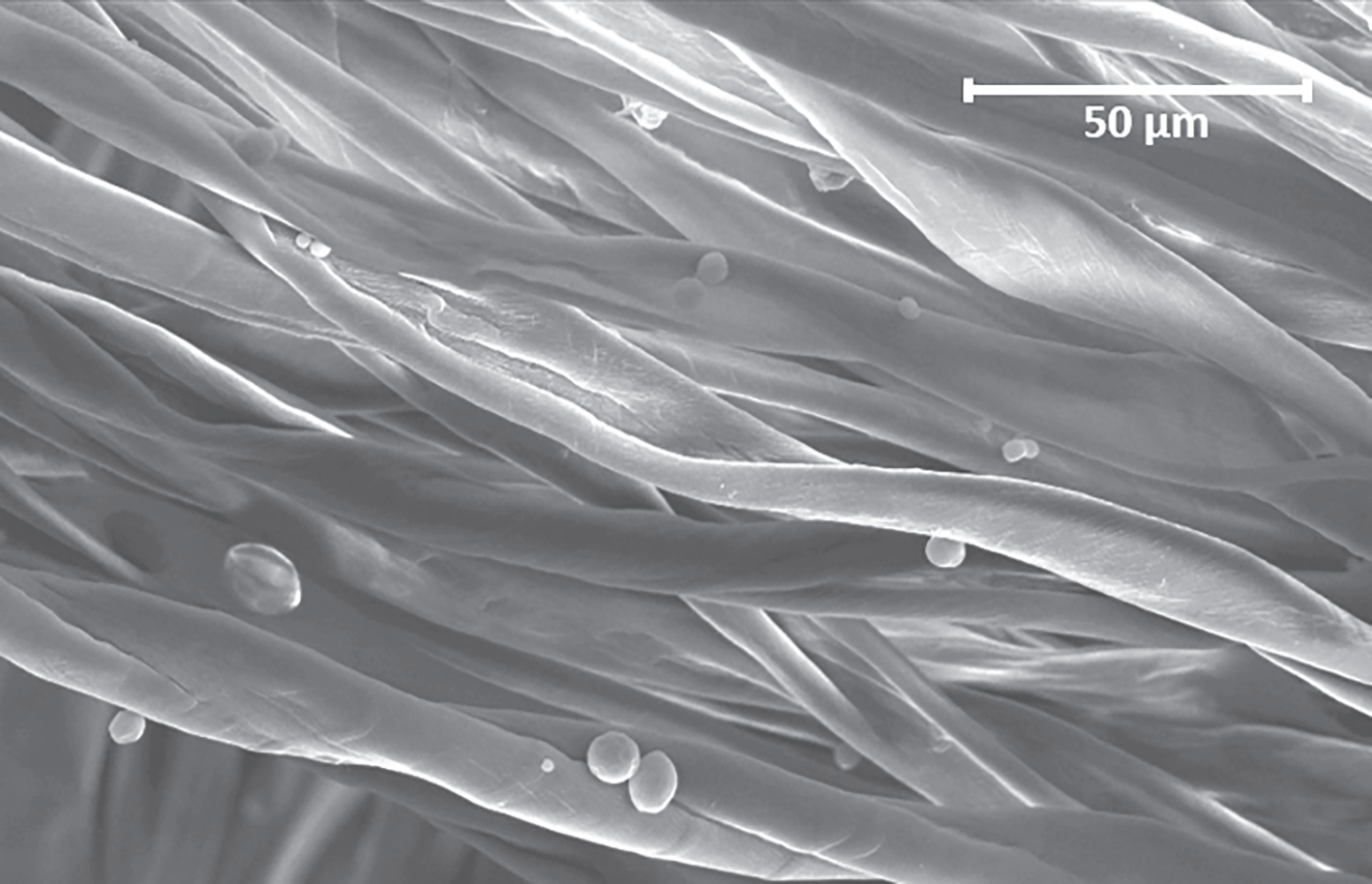
Scanning electron microscope (SEM) image of a cotton net fabric containing microcapsules.

Microcapsules containing delta-undecalactone were produced by a solvent evaporation technique using an oil-in-water emulsion [48,49]. We selected as shell material poly(lactic acid), a biodegradable polymer that is non-toxic, environmentally friendly and that has been thoroughly studied for its use in encapsulating hydrophobic drugs [50]. The core material was delta-undecalactone, which was slowly released by diffusion through the porous shell. The microcapsules consisted of 30% wt. delta-undecalatone (determined by thermogravimetric analysis) and were applied onto 100% cotton net fabric that was especially designed for this purpose (Leno structure, 65 g/m^2^, provided by Utexbel, Belgium). The application on the substrate was performed by padding, thereby obtaining a wet pickup of 67%, and the product was dried at 110°C. The result was a repellent-impregnated fabric containing 2.18 g dry microcapsules per m^2^. Fig 6 shows a scanning electron microscope (SEM) image of this fabric, confirming the presence of the microcapsules.

### Laboratory experiment

#### Mosquitoes

The mosquitoes (An. coluzzii, formerly An. gambiae s.s. form M) used in the laboratory experiment were reared in climate chambers at the Laboratory of Entomology of Wageningen University, The Netherlands. The original population was collected in Suakoko, Liberia, in 1987 (by courtesy of Prof M. Coluzzi).

Mosquitoes were kept under 12:12 h photo:scotophase at a temperature of 27 ± 1°C and relative humidity (RH) of 80 ± 5%. Adults were kept in 30 × 30 × 30 cm gauze wire cages and were given access to human blood through a Parafilm membrane every other day. Blood was obtained from a blood bank (Sanquin Blood Supply Foundation, Nijmegen, The Netherlands). A 6% glucose solution in water was available *ad libitum*. Eggs were laid on wet filter paper and then placed in a plastic tray with tap water for emergence. Larvae were fed on Liquifry No 1 (Interpet, UK) for the first three days and then with TetraMin baby fish food (Tetra, Germany) until they reached the pupal stage. Pupae were collected from the trays using a vacuum system and placed into a plastic cup filled with tap water for emergence.

The mosquitoes intended for the experiments were placed in separate cages as pupae. They had access to a 6% glucose solution but did not receive blood meals. The day preceding the experiment, 5–8 day old female mosquitoes were placed in release cages with access to tap water in cotton wool until the experiment. Both experiments took place during the last four hours of the scotophase, a period during which *An*. *gambiae* females are highly responsive to host odours [51].

#### Bioassay

The bioassay was set up in a climate-controlled room at constant air temperature (24 ± 1°C) and RH between 60 and 75%. During the experiments these parameters were monitored using a Tinyview data logger with display. Central to the bioassay was a landing stage to which mosquitoes were attracted. It consisted of a heated circular plateau (Ø 15 cm) that held the five-compound odour blend and was positioned underneath the gauze bottom of a flight chamber. The temperature at the centre of the landing stage was kept at 34 ± 2°C, comparable to the temperature of human skin, causing the mosquitoes to land and probe with their proboscis through the gauze in search of a blood-host.

#### Measuring repellence

A 15 cm x 15 cm cutting of the repellent-treated fabric was compared to an identical cutting of untreated fabric. The fabric was laid down on the bottom of the flight chamber, over the landing stage. Repellence was measured by releasing ten female mosquitoes into the flight chamber. After one min. of acclimatization time, the number of landings on the fabric covering the landing stage was counted during eight min. A landing was defined as the total period during which a mosquito maintained contact with the landing stage. Walking/hopping around on the landing stage as well as short (< 1 s) take offs immediately followed by landing again were included in one landing. A new landing was recorded when a mosquito had left the stage for more than 1 s before landing again. Landings shorter than 1 s during which no probing took place were ignored.

#### Design and data analysis

The treated and the control fabric were tested eight times, with four replicates per day of each, in random order, during two subsequent days. The tests were performed within a week after the treatment had taken place and were repeated after one, three and six months. In between tests, the fabric was stored at 4°C in a refrigerator. IBM SPSS Statistics 19 was used for data analysis. For the different moments in time, the number of landings on the treated fabric was compared to the control. A Shapiro-Wilk test was used to test for normality. T-tests were performed to determine significant reductions at α = 0.05.

### Field experiment

#### Study site

Kigoche village is located in Kisumu county in western Kenya. It lies adjacent to the Ahero rice irrigation scheme (00°08′19″S, 34°55′50″E) at an altitude of 1,160 m above sea level [26]. Kigoche has an average annual rainfall of 1,000–1,800 mm and an average RH of 65%. Mean annual temperatures in the area vary between 17°C and 32°C. Rice cultivation is the main occupation of the inhabitants. Most houses in the village are mud-walled with open eaves, have corrugated iron-sheet roofs, no ceiling and are either single- or double- roomed. Eaves, about 20 cm wide, increase ventilation in the houses and form the predominant entry points for mosquitoes [28,52]. Malaria caused by *Plasmodium falciparum* is endemic in the village. The area experiences a long rainy season between April and June and a short rainy season in October—November. During these periods, mosquito breeding sites proliferate, and mosquito populations rapidly increase in size. The domestic animal population comprises cattle, goats, sheep, chickens, ducks, dogs and cats, with cattle being most abundant. The main staple food is maize. Rice is primarily grown as a cash crop.

#### Houses

Eight traditional, mud-walled houses were selected for the baseline study (see below). The minimum distance between any two selected houses was 30 m, but other (unselected) houses were present around and in between. Based on the mosquito catches during the baseline experiment, four out of the eight houses were selected for the subsequent push-pull experiment.

#### Measuring house entry

Mosquitoes were attracted into a house by a volunteer sleeping under an untreated bed net. Eight male volunteers were recruited to sleep in the houses, one person per house. There were no other people sleeping in the house. The CDC light trap was installed at the foot end of the bed, with the top cover hanging approximately 15 cm above the matrass. The light of the trap was disabled, in order to collect only mosquitoes attracted by the volunteer. Power for the fan was supplied by a 6 V dry cell battery. Vaseline petroleum jelly was applied to the string from which the trap hung down, preventing ants from reaching the mosquitoes in the trap. The eight volunteers rotated amongst the houses. Each night the collection of mosquitoes started at 19:30 h and stopped at 6:30 h in the morning.

Trapped mosquitoes were killed in a freezer and morphologically identified. Culicine mosquitoes were identified to genus level and anophelines were divided into *An*. *funestus* sensu lato (s.l.), *An*. *gambiae* s.l. and other *Anopheles* spp. Individual *An*. *funestus* s.l. and *An*. *gambiae* s.l. mosquitoes were placed into 2 ml Eppendorf tubes with silica gel and a piece of cotton wool to be further identified with a polymerase chain reaction (PCR) [21–23]. The abdominal status of female mosquitoes was categorized as unfed, blood-fed or gravid.

#### Interventions

The four treatments that were tested during the field experiment were: (i) the control treatment, in which a house received neither repellent-impregnated fabric nor an attractant-baited trap. (ii) a push-only treatment in which only the repellent-impregnated fabric was installed, (iii) a pull-only treatment in which an attractant-baited trap was installed outside the house and (iv) a push-pull treatment in which both the repellent-impregnated fabric and the attractant-baited trap were in place.

**Fig 7:**
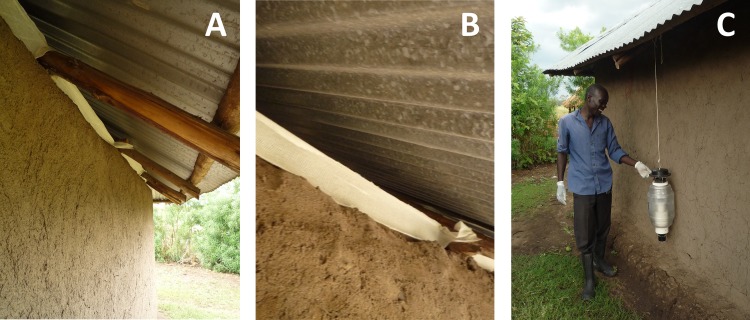
The components of the push-pull system. Panels A and B: The 10 cm wide strip of fabric as it was applied inside the eave, around the full circumference of the house. Panel C: The attractant baited MM-X trap as it was installed outside the house.

The repellent was released from a 10 cm wide strip of the fabric described above, which was applied inside the eave, around the full circumference of the house (Fig 7A and 7B). The strip was stretched in the lower part of the eave, closing off only the bottom 10 cm but leaving ample space for mosquitoes to enter the house. The control and pull-only treatments received an untreated strip of fabric that was applied the same way as the treated fabric used in the push and push-pull treatments. Strips remained in place over the entire study. See Table 4 for a comprehensive overview of the presence/absence of the specific elements during the treatments.

**Table 4:** Overview of which push and pull elements were present during the various interventions.

Intervention	Fabric in eave	MMX trap outside
Control	untreated	No
Push only	treated	No
Pull only	untreated	Yes
Push-pull	treated	Yes

The attractant-baited traps were of the Mosquito Magnet X (MM-X) type [53,54], baited with the five-compound blend described above and CO_2_ produced by the fermentation of molasses by yeast [55,56]. Traps were installed outside, with the odour outlet positioned at 15 cm above ground level (Fig 7C) [28]. A 12V battery provided power for the MM-X traps. Surgical gloves were worn when handling the traps, to avoid contamination with human odour.

#### Study design

Data from a baseline study allowed us to correct for initial differences between the houses in terms of mosquito entry by using a difference-in-differences method rather than a simple cross-sectional comparison to estimate the impact of the interventions [57]. The baseline study was conducted during eight subsequent nights (a full rotation of all volunteers), to determine the house entry of mosquitoes for eight different houses. Hereafter, four houses were selected based on the mean number of mosquitoes caught and the variation between the different nights (see details in the results section). Treatments were randomly assigned to the selected houses.

Immediately following the baseline study, the push-pull experiment ran for five subsequent weeks. During the first two rounds of eight nights, sampling took place every night ((n = 8) * 2). For the last three weeks, sampling took place three nights a week ((n = 3) * 3). House entry was measured by CDC trap catches, as during the baseline study. The differences between the mean indoor catches were corrected for by subtracting the mean trap catches of the baseline study from the data obtained during the intervention phase. For a conservative estimate, we used the pooled variance of the intervention phase data, which was larger than the pooled variance of the baseline data, for further testing. The mean trap catches of the different interventions were compared with the control treatment using a General Linear Model (GLM) followed by Dunnet’s post-hoc test. Testing was one-sided (treatment < control) with overall α = 0.05. IBM SPSS Statistics 19 was used to generate GLMs and post-hoc tests.

### Ethics statement

This study was part of a series of studies that were approved by the ethical review committee of the Kenya Medical Research Institute (KEMRI/RES/7/3/1). The purpose and procedures of the study were explained to local leaders, household heads and volunteers before seeking permission to carry out the study. Volunteers and house owners were informed about the nature of the study and consented after having read and understood the protocol of the study prior to signing two copies of the written consent form approved by the ethics committee of KEMRI. One of the copies was kept by the participant while the second one was retained for the project record. During the experiment there was daily communication with the volunteers, who had continuous access to artemisinin combination therapy (ACT) in case of infection with malaria. The individual in Fig 7C has provided specific permission (as outlined in the PLOS consent form) for his picture to be used in this publication.

### Malaria transmission model

#### General description

The deterministic and static model by Okumu et al. [24] describes and quantifies the most essential activities of malaria mosquitoes in the process of malaria transmission. Over 70 parameters describing these activities are included in the model, roughly captured in ecological parameters, intervention parameters and parameters that are derived from combinations of those. The model assumes that the population is homogeneously exposed to mosquitoes, no cumulative or time effects are considered and biting finds place exclusively indoors and during the night. See [24] for full details concerning the parameterization of all variables and literature references. Using the entomological inoculation rate (EIR, the average number of infectious bites received by a person in a year [58]) as a proxy, we determined the effect of a possible push-pull intervention on malaria transmission for a number of scenarios.

#### Model settings

We used the default settings of the model, with exceptions for the following parameters:

Bed net use (Ch) is set at 67%, i.e. 2/3 of the population is assumed to possess a bed net and sleep under it. The model acknowledges the dual efficacy of ITNs, using one parameter to express the excess diversion (θD) and another parameter to express the excess mortality (θm) that a mosquito experiences upon attacking a human being sleeping under an ITN. The latter parameter is adjusted in a second series of scenarios that explored the effect of pyrethroid resistance (see below).

In order to include the influence of repellent-induced house entry reduction (push efficacy) on the EIR, we interpreted this effect as a human being less available for a blood meal. Push efficacy is thus represented by reduced availability of all humans (those with and those without a bed net) for blood meals. Thus, when the efficacy of the push (ps) is defined as the fraction of mosquitoes that is prevented from entering the house by the repellent barrier, then the availability of humans (ah) decreases through ah * (1-ps), which results in the relative availability of humans (rah). We used rah instead of ah in all scenarios, considering house entry reduction of 0–100% (10, this paper). In the absence of the push-intervention ps = 0, thus rah = ah.

We used the relative attractiveness of the attractant-baited traps (λt) as a measure for the efficacy of the pull. The efficacy of the pull is the attractiveness of the trap compared to that of a human being, thus when λt = 1, the trap is as attractive as a human being. We considered values of 0, 0.5, 1 and 2 for λt [25,26]. In the absence of the pull intervention λt is set to 0. Availability of odour-baited traps, which in the original model is linked to human availability, was set to 0.0012, its default value, identical to that of a human being in the absence of the push intervention. Each household, assumed to consist of six people, is supposed to possess one odour-baited trap. Therefore, using the default number of people (1000), the number of odour-baited traps is set to 167.

To explore the effects of possible push-pull interventions in a situation where pyrethroid resistance is widespread, the excess mortality that a mosquito experiences upon attacking a human being sleeping under a bed net was reduced in a second series of scenarios. A recent review by Strode et al. [59] addressing the risk difference, in terms of mortality, for a mosquito attacking someone sleeping under a non-treated net versus someone sleeping under an ITN, allowed us to reliably estimate this parameter, which we set to 0.4 (from 0.7 in the default scenarios) to mimic a high resistance situation.

## Supporting Information

S1 Dataset Raw data of the laboratory experiment. Provided in adherence to the PLOS policy to make all data underlying the findings described in this manuscript fully available.
(XLSX)

S2 Dataset Raw data of the field experiment. Provided in adherence to the PLOS policy to make all data underlying the findings described in this manuscript fully available.
(XLSX)

S1 Fig Model simulations showing the entomological inoculation rate (EIR) as a function of different levels of pull efficacy. Pull efficacy is expressed as the relative attractiveness of the trap, compared to a human being. Push efficacy is expressed as the percentage of house entry reduction. In this scenario mosquitoes are fully susceptible to insecticides.
(TIF)

S2 Fig Model simulations of a scenario in which mosquitoes are highly resistant against insecticides. Shown is the entomological inoculation rate (EIR) as a function of different levels of pull efficacy. Pull efficacy is expressed as the relative attractiveness of the trap, compared to a human being. Push efficacy is expressed as the percentage of house entry reduction.
(TIF)

S1 Table Mean catches of *Anopheles funestus* mosquitoes for the different interventions. For the baseline data n = 8 (n = 7 for house 3) and for the intervention data n = 25.
(DOCX)

S2 Table Mean catches of *Anopheles gambiae s*.*l*. mosquitoes for the different interventions. For the baseline data n = 8 (n = 7 for house 3) and for the intervention data n = 25.
(DOCX)
