# Supplementary figures and images for: Field Evaluation of a Push-Pull System to Reduce Malaria Transmission
Source: PLoS One. 2015 Apr 29;10(4):e0123415. doi: 10.1371/journal.pone.0123415 (PMC4414508; doi:10.1371/journal.pone.0123415)

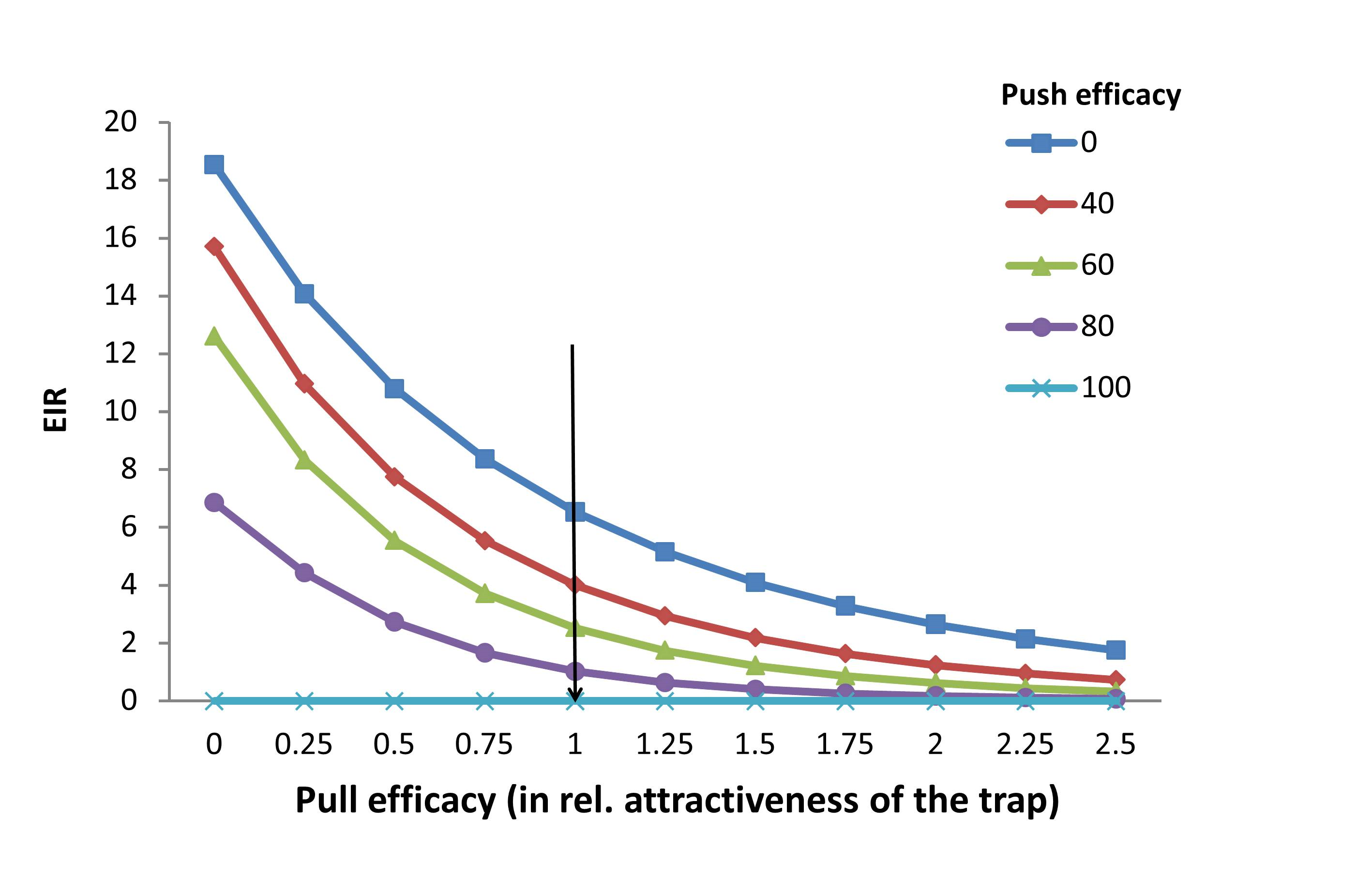

Supplement: S1 Fig — Pull efficacy is expressed as the relative attractiveness of the trap, compared to a human being. Push efficacy is expressed as the percentage of house entry reduction. In this scenario mosquitoes are fully susceptible to insecticides. (TIF) [file pone.0123415.s003.tif]

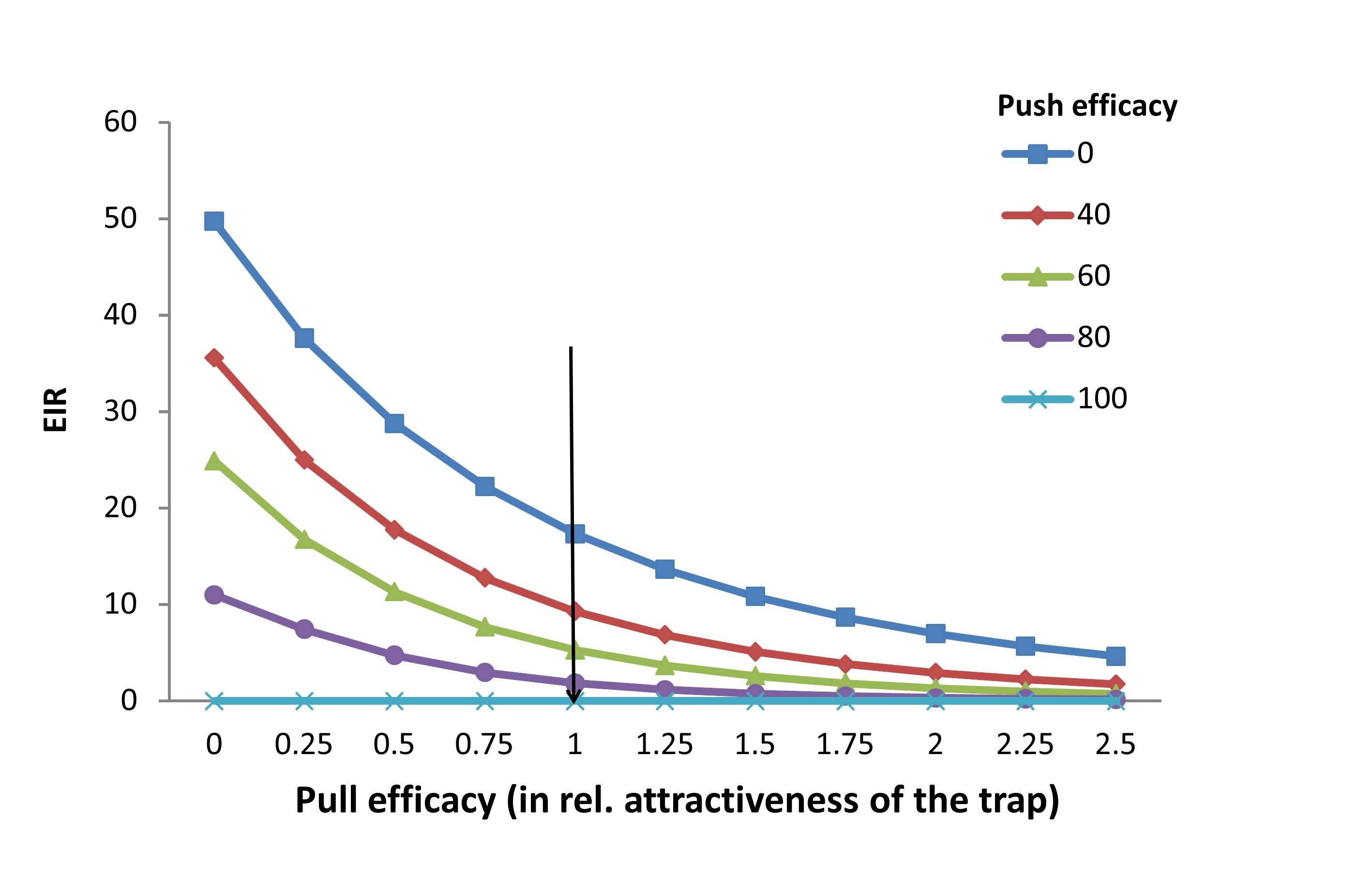

Supplement: S2 Fig — Shown is the entomological inoculation rate (EIR) as a function of different levels of pull efficacy. Pull efficacy is expressed as the relative attractiveness of the trap, compared to a human being. Push efficacy is expressed as the percentage of house entry reduction. (TIF) [file pone.0123415.s004.tif]
