# Supplementary material for: Field Evaluation of a Push-Pull System to Reduce Malaria Transmission
Source: PLoS One. 2015 Apr 29;10(4):e0123415. doi: 10.1371/journal.pone.0123415 (PMC4414508; doi:10.1371/journal.pone.0123415)
Supplement: S1 Table — For the baseline data n = 8 (n = 7 for house 3) and for the intervention data n = 25. (DOCX) [file pone.0123415.s005.docx]

**Table S1. Mean catches of *Anopheles funestus* mosquitoes for the different interventions.** For the baseline data n = 8 (n = 7 for house 3) and for the intervention data n = 25.

| **Intervention** | **House** | **Baseline** | **Intervention** | **Difference** | **Difference (%)** | **Impact** |
| --- | --- | --- | --- | --- | --- | --- |
| Control | 4 | 12.75 | 13.12 | 0.37 | 2.9% | n/a |
| Push | 5 | 10.13 | 4.40 | -5.73 | -56.6% | -59.5% |
| Pull | 3 | 8.57 | 4.76 | -3.81 | -44.5% | -47.4% |
| Push-pull | 1 | 14.00 | 7.56 | -6.44 | -46.0% | -48.9% |
