# Supplementary material for: Field Evaluation of a Push-Pull System to Reduce Malaria Transmission
Source: PLoS One. 2015 Apr 29;10(4):e0123415. doi: 10.1371/journal.pone.0123415 (PMC4414508; doi:10.1371/journal.pone.0123415)
Supplement: S2 Table — For the baseline data n = 8 (n = 7 for house 3) and for the intervention data n = 25. (DOCX) [file pone.0123415.s006.docx]

**Table S2. Mean catches of *Anopheles gambiae s.l.* mosquitoes for the different interventions.** For the baseline data n = 8 (n = 7 for house 3) and for the intervention data n = 25.

| **Intervention** | **House** | **Baseline** | **Intervention** | **Difference** | **Difference (%)** | **Impact** |
| --- | --- | --- | --- | --- | --- | --- |
| Control | 4 | 2.88 | 2.00 | -0.88 | -30.6% | n/a |
| Push | 5 | 3.50 | 1.28 | -2.22 | -63.4% | -32.9% |
| Pull | 3 | 2.29 | 0.92 | -1.37 | -59.8% | -29.3% |
| Push-pull | 1 | 7.75 | 2.36 | -5.39 | -69.5% | -39.0% |
